# Site Selection of Urban Parks Based on Fuzzy-Analytic Hierarchy Process (F-AHP): A Case Study of Nanjing, China

**DOI:** 10.3390/ijerph192013159

**Published:** 2022-10-13

**Authors:** Chenying Li, Tiantian Zhang, Xi Wang, Zefeng Lian

**Affiliations:** 1 Department of Landscape Technology, Suzhou Agricultural Vocational and Technical College, Suzhou 215008, China; 2 Department of Landscape Architecture, School of Architecture, Soochow University, Suzhou 215006, China; 3 Department of Architecture, Nanjing University, Nanjing 210024, China; 4 Department of Landscape Architecture, Suzhou University of Science and Technology, Suzhou 215011, China

**Keywords:** multi-criteria decision analysis (MCDA), sustainable development, triangular fuzzy number, once-at-a-time (OAT)

## Abstract

The scientific siting of urban parks is critical for sustainable urban environment development, and this study aimed to identify suitable areas for future urban parks in Nanjing, China. This study has integrated geographic information systems (GIS) and fuzzy hierarchical analysis (F-AHP) in order to evaluate the suitability of the site selection of urban parks in Nanjing, China. Different physical, natural, environmental, accessibility, and human activity factors were evaluated in order to assess the suitability of a park site. The results revealed that 5% were highly suitable for urban park site selection, 36% were more suitable, 32% were moderately suitable, 19% were less suitable, and 8% were unsuitable for urban park site selection. The findings suggest that the areas that are highly suitable for urban park placement are located in the western and eastern parts of Nanjing. Carbon storage was the most important factor in the suitability of urban park site selection, followed by the normalized difference vegetation index (NDVI) and the heat-island effect. The methodology that has been adopted in this study helps to improve the methodological framework of combining F-AHP and GIS; in addition, generating urban park site selection maps assists planners and decision-makers in making scientific site selection decisions.

## 1. Introduction

With continued global urbanization and rapid economic developments, meeting the production and living needs of the population that is gathered in cities will inevitably have a severe effect on the ecological environment [1,2,3,4], causing a series of urban environmental problems, such as traffic congestion [5,6,7], environmental degradation [8,9], vegetation destruction, and air pollution [10,11,12,13,14,15,16]. Urban parks are essential for enhancing city air quality, lowering noise pollution in urban areas, and fostering sustainable urban growth [17,18,19]. The current global COVID-19 outbreak has raised the demand for urban green space [20,21]. The park areas and the ecological requirements of many towns hardly meet the various needs of their populations. New urban parks must be built in order to resolve the supply and demand issue. Therefore, an urban park site assessment model must be created in order to help determine the most suitable future urban park locations by utilizing decision-support tools. This will help to address these concerns and to encourage sustainable urban growth [22]. 

The use of geographic information systems (GIS) is the most popular technique for studying land-siting concerns [23]. It can successfully address the data visualization problem and boost the effectiveness of the data management and analysis. Multi-criteria decision analysis (MCDA) is a powerful technique that is used in order to assist with land-siting decisions. The analysis of several land-siting issues has shown the method’s scientific validity [24,25]. In terms of contemporary multi-objective land decision problems, the widespread use of MDCA in combination with GIS represents a substantial development in solving complex land placement challenges [26,27,28,29]. 

Previous studies have combined GIS with traditional AHP and fuzzy AHP (F-AHP), which are two main research methods that have been developed in the past decades, in order to deal with complex land-siting problems. A few researchers have investigated the utilization of genetic algorithms in park site selection [30,31]. The first method, which is traditional AHP [32,33,34,35,36,37,38,39], employs a weighted overlay tool within the GIS to produce the final suitable site selection map after using classical set theory to calculate the weights of each criterion. Olaniyi et al. (2018) [32] examined ecotourism site selection in national parks using the traditional AHP and mapped the land suitability of Okomu and Pendjari National Parks (ONP and PNP, respectively) by using a GIS weighted linear overlay. The results showed that in ONP, most hotspots were in unsuitable sites for ecotourism, but in PNP, all of them were on moderately suitable land. In another study, Ristić et al. (2018) [33] integrated MDCA (AHP) and GIS techniques in order to create a land sustainability site selection model for Sara Mountain National Park. According to the findings, only 24% of the land in the protected region was eligible for land sustainability site selection, while 36% of the land was not. Georgiou and Skarlatos (2016) [34] used the traditional AHP to estimate the weights of the following four factors: environmental, economic, social, and technological, and then combined them with a GIS in order to achieve data overlay analysis in the optimal solar park site selection study. The results suggested that only 3% of the research area was suitable for a solar park and that more than 80% of the region was completely unsuitable. Zhang et al. (2019) [35] mapped the viability of urban park sites utilizing AHP and GIS with the land construction feasibility, the urban heat-island effect, the air pollution, the park accessibility, the radius of urban disaster prevention and avoidance services, and the spatial distribution of the population as factor input layers. They ultimately chose one large park and seven small parks.

The second method, which is the fuzzy AHP [30,40,41], employs fuzzy set theory in order to categorize the parameters according to their significance and then determines the weight of each parameter in order to generate the park suitability site selection areas via GIS overlay.

Pakfetrat et al. (2020) [30] selected the following four factors: physical, environmental, social, and economic, in order to establish a regional park site selection model, used fuzzy triangular numbers to determine the layer weights of each factor, and finally realized the comprehensive overlay of each element through GIS. The results demonstrated that fuzzy hierarchical analysis is appropriate for locating urban parks.

The fuzzy analytic hierarchy process (F-AHP) was used in Zabihi et al.’s (2020) ecotourism suitability study [40] in order to determine the influence of physical, natural, environmental, and socio-economic factors on identifying ecotourism sites. The study revealed that 16.6% (251 km^2^) of the study area was highly suitable for ecotourism site selection.

Determining the weights that were allocated to each criterion is a crucial step in solving the land-siting problem, as shown by an overview of the two primary research methods that were mentioned earlier. Several criteria, and the allocation of various weights, complicate the evaluation procedure in the land-siting study. Even though multiple methods can be used in order to determine the weights of criteria, the classic AHP is regarded as one of the most notable approaches to multi-criteria decision making. Numerous researchers have discovered that selecting the decision model, the weighting factors, the data, and the human judgment errors can contribute to the internal uncertainty features of multi-criteria decision making. The decision process of the traditional AHP method ignores the uncertainties that have been mentioned above, resulting in incorrect multi-criteria decision making and distorted evaluation outcomes. The limitations of the conventional AHP method process that are related to the data uncertainty and the subjective judgment uncertainty are well compensated [42] by the fuzzy-analytic hierarchy process [43]. 

In addition, a review of the relevant literature on land siting has concluded that two main types of fuzzy techniques could be applied in siting studies. One is to determine the factor layer weights using fuzzy techniques, and the other is to classify the criteria by defining the different types of fuzzy functions. The most recent research methodologies that have been used in the siting literature have applied only one fuzzy strategy [41,44,45,46,47].

Therefore, most of the current research on urban park siting has utilized a combined AHP–GIS approach, and a few studies have utilized a combination of fuzzy AHP and GIS for analysis. However, the fuzzy technique was utilized only once in the urban park site selection study, either in the weight determination or the GIS standard fuzzy classification. It does not use fuzzy techniques in both weight determination and fuzzy classification of GIS data. This study proposed a new approach to combine GIS with F-AHP. Although many studies [40,48,49] have shown this approach to be more efficient and adaptable than the other approaches, we applied fuzzy theory to both the weighted and the standard classification processes for urban park site selection in our study. 

A review of the literature on sustainable urban planning has concluded that sustainable transportation [50,51], environmental protection and restoration [52,53], renewable energy and waste management [54,55,56], environmental justice and social equity [57,58], economic development [59,60], sustainable land use [61,62], and urban design [63,64] are the current hotspots for sustainable cities [65]. Some scholars have carried out in-depth research on restoring parks and greenways in vulnerable urban areas in the direction of sustainable land use and urban design. Still, few scholars have studied the sustainable development of urban parks. Urban parkland use plays a vital role in preventing urban sprawl and in mitigating the negative impact of cities on the environment [66,67]. Therefore, we added environmental factors to the site selection of urban parks. The normalized difference vegetation index (NDVI), the heat-island effect, the air pollution, and the carbon storage were included as urban park site selection criteria for the first time. These standards help us to avoid the construction of urban parks at the expense of the natural environment and to guarantee the sustainability of the urban environment.

**Figure 1:**
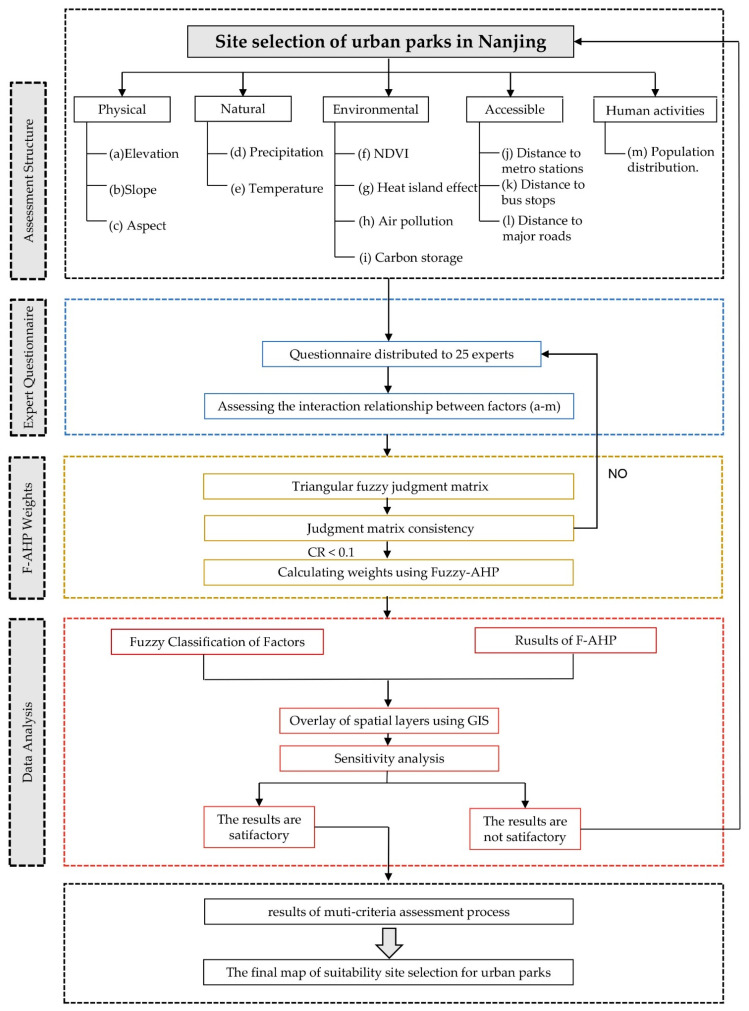
Flow Chart of urban park site selection evaluation in Nanjing.

Overall, the main objective of this study was to use F-AHP combined with GIS to prepare an urban siting map of Nanjing and to establish suitable areas for siting urban parks in Nanjing. This study integrated geographic information systems (GIS) and fuzzy hierarchical analysis (F-AHP) (Figure 1). Applying the fuzzy technique to the AHP weight determination and GIS indicator classification process minimized the limitations of the existing single fuzzy technique that were caused by the uncertainty of the indicator data and the inaccurate classification. In addition, including the ecological and the environmental elements in the urban park site selection criteria filled a gap in the study of the sustainable development of urban park sites. We developed an urban park siting model for the Nanjing area by integrating the F-AHP model and testing the model sensitivity by using a global sensitivity analysis that was based on the OAT technique. Finally, we compiled a map of the urban park site selection for the Nanjing area. The methodology that was adopted in this study helps to improve the methodological framework of combining F-AHP and GIS; in addition, generating urban park site selection maps assists planners and decision-makers in making scientific site selection decisions.

## 2. Materials and Methods

### 2.1. Study Area and Data Sources

**Figure 2:**
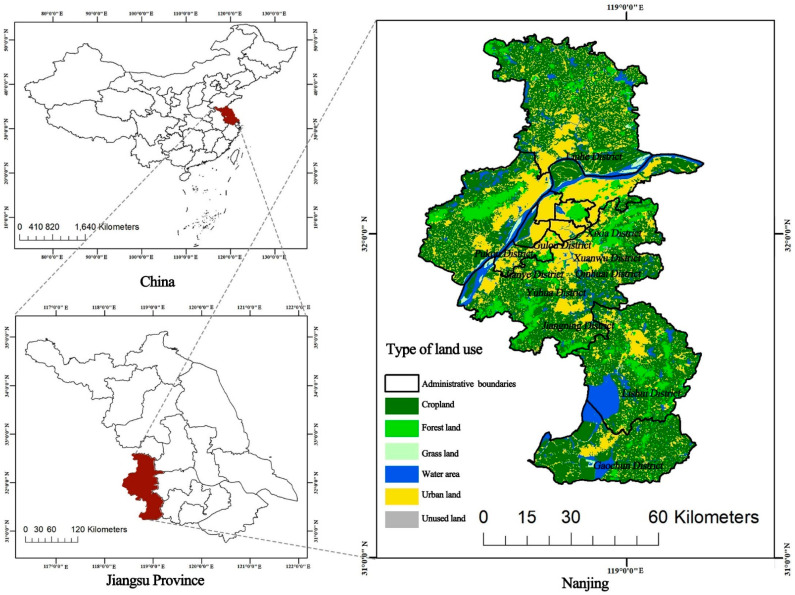
The study area of the Nanjing.

The area of this study was in Nanjing, China (Figure 2), which is located in eastern China, in the middle and lower reaches of the Yangtze River, with geographical coordinates marked between 31°14′ and 32°37′ N latitude and 118°22′ and 119°14′ E longitude. Nanjing has jurisdiction over 11 districts, with a built-up area of 868.28 km^2^, as of 2020, with many Yangtze River rocks, rich water, and various other coexisting geomorphic units. More than 11% of Nanjing’s area is covered by water. As of 1 November 2020, Nanjing had a population of over 9.3 million and an urbanization rate of 86.8%. The increased urbanization rate has vastly increased residents’ demand for urban parks. Nanjing, which is a world-class second-tier city [68], the sub-center of the Yangtze River Delta and China’s largest globalized metropolitan region [69], will have 1001 parks by 2035, according to the Nanjing Park Layout Plan (2017–2035). How to scientifically plan and site urban parks to meet the needs of the residents has become a practical problem that needs to be solved. 

### 2.2. Determination of Urban Park Site Selection Criteria and Spatial Data Acquisition

#### 2.2.1. Urban Park Site Selection Criteria

The urban park is a unique landscape category among urban blue–green space types [70], offering leisure and recreation [71,72,73], alleviating the heat-island effect [74,75,76], protecting biodiversity [77], and improving urban climate [78]. The ecological functions of urban parks, such as air purification and microclimate improvement, which are highly pertinent to the study objectives, were also considered, in addition to the essential functions of parks, such as the accessibility of the parks and their distance from the population centers [79]. This type of land-siting scheme, with a focus on nature, not only protects the original ecological environment, but also reduces the capital investment in green plant transplanting, promoting the implementation of the simplification action of urban park landscape development, and having a high social and ecological value [80,81].

Based on literature reviews [30,35,82,83,84], we divided the urban park site selection criteria into the following five categories: physical factors, natural factors, environmental factors, accessibility factors, and human activity. We used elevation, slope, and aspect as physical factors; precipitation and temperature as natural factors; normalized difference vegetation index (NDVI), heat-island effect, air pollution, and carbon storage as environmental factors; distance to metro stations, bus stops, and major roads as accessibility factors; and spatial distribution of the population as human activity factors. 

**Figure 3:**
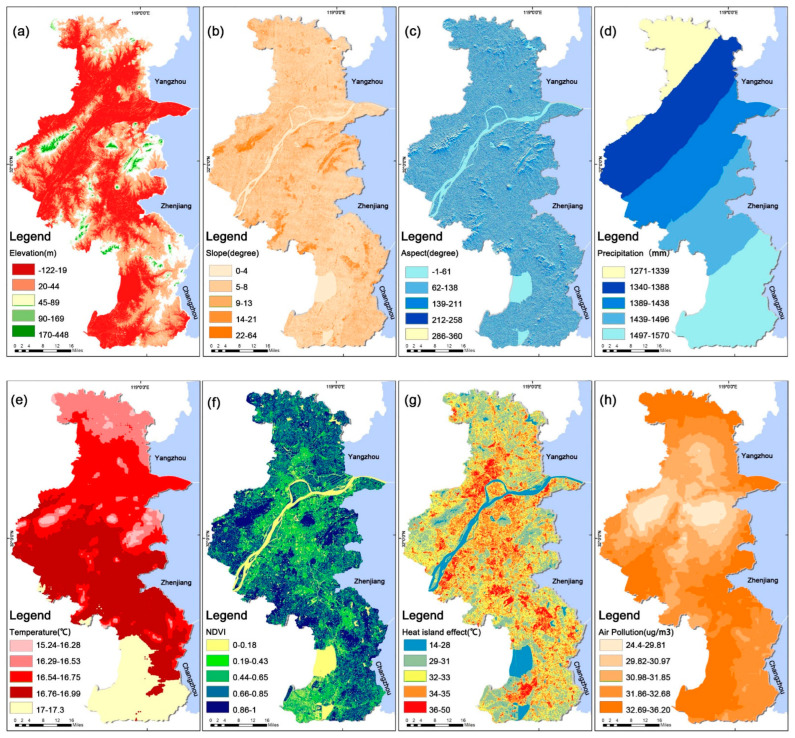

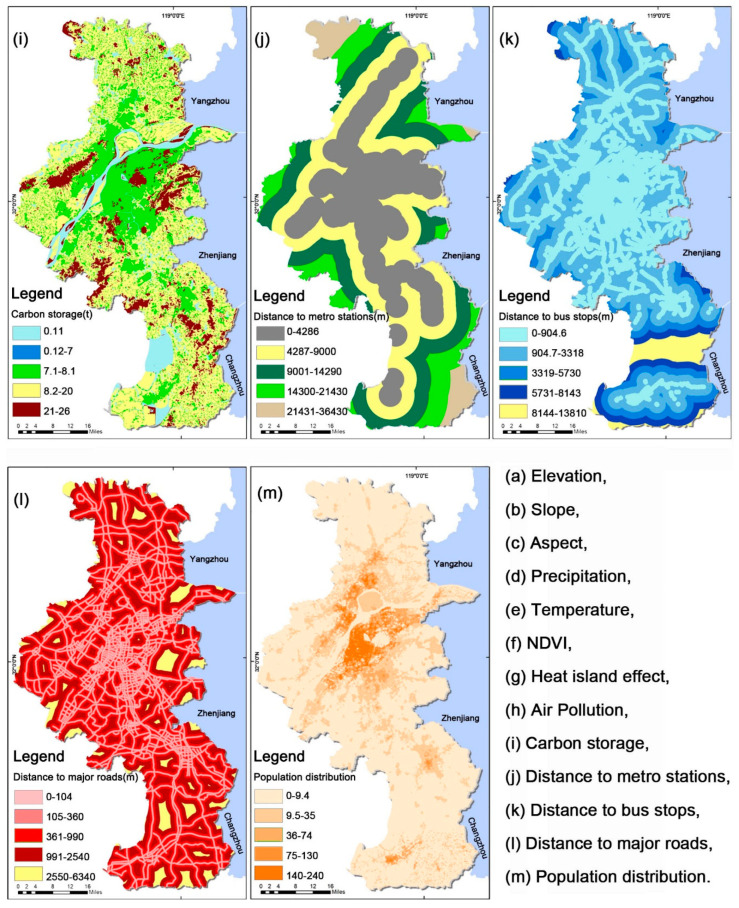
Factors relevant for determining the location of urban parks in Nanjing, China.

Among the urban land types, parcels with plains or near plains, gentle slope changes, and south- and east-facing slopes have the highest physical property suitability values for urban park siting [48,85,86]. Urban land types with dramatic slope changes pose health risks to residents due to increased development design difficulties and soil erosion on slopes when they are selected as sites for urban park development [87,88]. In addition, the north, northeast, and northwest directions receive much shorter sunlight times than the southern parcels, limiting the diversity of plant life in the park [89] and adversely affecting the park microclimate changes [90] (Figure 3a–c).

The natural factors of average annual temperature and precipitation can effectively reflect the natural environment within the study area, and the suitable temperature [91] and precipitation [92] are also essential factors in determining the park’s location (Figure 3d,e).

The normalized difference vegetation index (NDVI) is not affected by cloudiness, atmosphere, or other factors. The larger the value of this index, the more robust the photosynthesis and transpiration of the plants, and the more suitable for residents to rest [92]. The heat-island effect (Figure 3g) reflects the urban surface temperature distribution. Areas with a high heat-island effect have higher surface temperatures, therefore, the economic costs required to site a park on such an area are higher and the ecological benefits harvested are lower [93]. PM2.5 data are an essential indicator of air pollution within a particular area. The higher the PM2.5, the heavier the air pollution in the land area, which is not conducive to park site selection and construction. The carbon storage (developed by the US INVEST model) reflects the amount of carbon that is stored in the study area. Total carbon storage is the sum of the above-ground carbon storage, below-ground carbon storage, carbon and water storage in the soil, and organic matter carbon storage. High total carbon storage indicates the higher ecological nature of the study section area (Figure 3i) [94]. 

The spatial distribution of human activities in the area is studied in urban park site selection [77] to understand sites that are less affected by human activities that have more spatial population distribution. The effect of human activity intensity on park ecology is enormous, so the park becomes less ecologically effective with a more spatially distributed population (Figure 3m). 

When choosing park locations, accessibility to transportation is crucial. Due to their proximity to the city’s primary transportation network system, locations with metro stations, bus stops, and major city roads offer greater accessibility to a city park for recreational activities (Figure 3j–l) [95].

#### 2.2.2. Geospatial Data Acquisition

Plots near metro stations, bus stops, and major urban roads are close to the leading urban transportation network, decreasing travel time and cost and facilitating access to a city park for recreational activities. The elevation data used in the study were obtained from the 2022 Nanjing, China DEM data of the geospatial data cloud platform, numbered ASTGTM2_N31E118 and ASTGTM2_N31E119. These data were used to generate slope and slope directions in ArcGIS. The precipitation data were derived from the multi-year average station dataset of the 2021 China Meteorological Science Data Sharing Network. The results were obtained by interpolating the longitude and latitude as independent variables and elevation as covariates in ANUSPLIN software, utilizing the thin-plate spline function interpolation method [96]. NDVI was taken from 30 m resolution Landsant8 satellite image data that was taken in September 2021 and numerically calculated using the satellite’s 4-band (infrared band) and 5-band (near-infrared band). The heat-island effect data were sourced from the thermal infrared sensor (TIRS 10 and TIRS 11) bands of the Landsat 8 satellite. The surface temperature was inverted using the Jimenez-Munoz single-channel algorithm model [97] to derive the study area’s spatial distribution of surface temperature. The data for metro stations, bus stops, and major roads were obtained from the 2022 Baidu Map Nanjing POI data; air pollution 2021 PM2.5 monitoring data were selected from conventional stations in Nanjing; and carbon storage capacity, which was an essential criterion for ecological value evaluation, was obtained from 30 m resolution Chinese land use remote sensing data in 2018. The total carbon storage in the study area was calculated using the Stanford University InVEST model (integrated valuation of ecosystem services and trade-offs). The population distribution was estimated using the 2013 DMSP/OLS (Defense Meteorological Satellite Program) satellite nighttime light data for the study area. 

All data layers have been normalized to a pixel size of 30 m × 30 m. GIS was used to calculate the suitability index of park sites for each raster cell to generate a raster map of urban park sites in Nanjing. 

### 2.3. Classification of Standards

It is crucial to categorize the geographical data of urban park site selection elements using classification criteria to analyze the suitability of park sites within a specific area. The two classification approaches for GIS raster images are classic deterministic and fuzzy classification. Deterministic classification is a classification in which an image element is defined as 1 if it entirely belongs to a particular class, and 0 if it does not. However, when processing remote sensing data, it is often found that certain image elements belong to more than one class. It is necessary to apply fuzzy classification to classify them into definite courses by using a specific fuzzy affiliation function. Fuzzy classification is widely used in geographic information research, where images of uncertain different geographic feature factors can be converted into affiliation values when performing classification. For example, in this study, no definite threshold was set to define whether the vegetation cover was high or low, so the fuzzy classification approach was more suitable for extracting information categories that could not be judged by “yes” or “no.” 

The fuzzy set and affiliation function are the mathematical basis of fuzzy classification. Fuzzy logic was developed initially by Zabihi (2020) [25], who defined a fuzzy set as “a class of objects with continuous affiliation [30]”.

(1) $$x\to u_{A}x,x\in U\;$$

A fuzzy set is defined as a number, *U_A_*(*U*) ∈ [0, 1], in the closed interval [0, 1] for any element, *x* ∈ *u*, on the domain of the argument. *U_A_*(*x*) is the affiliation function of the fuzzy set *A*. If the eigenvalue is *i*, then *U_A_*(*i*) is the subordination function of the fuzzy set *A* [98]. The affiliation degree of element *X* concerning the fuzzy set *A* can be expressed by the affiliation function *U_A_*(*x*). When *U_A_*(*x*) approaches 1, it means that the element *x* belongs to A higher degree, and when *U_A_*(*x*) approaches 0, it means that the element *x* belongs to A lower degree [99]. The cases of *U_A_*(*x*) = 1 and *U_A_*(*x*) = 0 represent that the element *x* belongs to the set *A* and the element *x* does not belong to the set *A*, respectively.

An affiliation function is a type of function that implements a specific element mapping to an appropriate degree of affiliation. The function can choose any curve form, such as linear, sigmoidal, j-shaped, Gaussian, or other more complex non-monotonic shapes [100,101,102]. In an urban park site selection study, it is impossible to use “yes” and “no” to classify the information categories uniformly, therefore, this study used fuzzy logic to organize the standard remote sensing images. The affiliation value of 1 represented the standard value of the most suitable urban park site, and the affiliation value of 0 represented the standard value of the least suitable urban park site. In addition, the fuzzy value corresponding to the standard was in the range of 0 to 1. 

This study identified linear and Gaussian functions as the affiliation functions for standard spatial data partitioning based on a literature review and expert empirical methods. The linear function is chosen when suitability rises with the standard value; the Gaussian function is chosen when suitability rises with the standard value but declines with the standard value after crossing the threshold (Table 1).

### 2.4. Weighting of Criteria

The triangular fuzzy hierarchical analysis, which can better support complex decision making than traditional hierarchical analysis, was used to determine the weights of the urban park site selection criteria [110]. The hierarchical analysis method is based on fuzzy triangular numbers when performing judgment matrix construction, which weakens the error of two-by-two comparison between standard factors and makes the weight calculation results more objective. According to the triangular fuzzy number principle, a set of real numbers *R* = (−∞,+∞) has a triangular fuzzy number *M* above it, and the affiliation function *U*_*M*_: *R* of *M* ranges from 0 to 1, satisfying the following equation [111]:

(2) $$u_{M}\left(x\right)=\{\begin{array}{c} \frac{1}{m-l}x-\frac{l}{m-1},x\in \left[1,m\right]\\ \frac{1}{m-u}x-\frac{u}{m-u},x\in \left[m,u\right]\\ 0,\mathrm{Others} \end{array}$$

**Figure 4:**
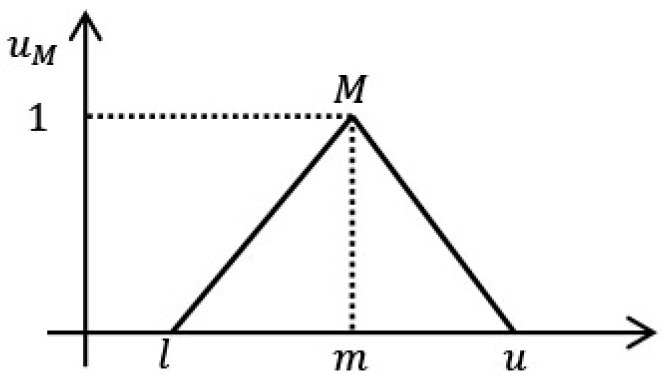
Triangular fuzzy number *M* = [*l*,*m*,*u*] and its relational function.

In Equation (2), the triangular fuzzy number is defined as l≤m≤u, where u is the upper limit and *l* is the lower limit, m is the median of the triangular fuzzy number *M*, and u−l represents the fuzzy interval of *M* (Figure 4). The interval range span refers to the expert’s degree of confidence in the results of the criterion judging. The more extensive the interval range, the lower the confidence level; the smaller the range, the higher the confidence level, which is calculated as follows [112]:

Step one involved establishing a triangular fuzzy judgment matrix. Experts were invited to construct the judgment matrix *A* = (*a_ij_*)*n* × *n* for the 13 sub-criteria that are listed in Table 1 using triangular fuzzy numbers. If the experts’ judgments were inconsistent, the average value would be considered as the final result. When *a_ij_* = [*l_ij_*,*m_ij_*,*u_ij_*] and *m_ij_* is the median of this triangular fuzzy number, the formula is as follows [113,114]:

(3) $$\;b_{ij}=\frac{1}{p}⊗\left(b_{ij}^{1}+b_{ij}^{1}+\cdots +b_{ij}^{p}\right),\;p=1,2,3\ldots P\;$$

The values of the fuzzy judgment matrix *a_ij_* are determined according to Table 2.

Step two involved a judgment matrix consistency test for the median *m_ij_*. By substituting the maximum characteristic root $\lambda_{max}$ of the median matrix *M* into Equation (4), the *CI* and *CR* values are obtained using Equation (5). When the judgment matrix *CI* = 0, it is complete consistency, and when *CR* < 0.1, the judgment matrix has satisfactory consistency [117].

(4) $$CI=\left(\lambda_{max}-n\right)/\left(n-1\right)$$

(5) CR=CI/RI 

Step three involved constructing the triangular fuzzy-based judgment factor matrix *E* (Equation (6)) and adjusting the judgment matrix so that its diagonal is 1 (Equation (7)) as follows:

(6) $$E={\left(e_{ij}\right)}_{n\times n}=\left[\begin{array}{cccc} 1 & 1-\frac{u_{12}-l_{12}}{2m_{12}} & \cdots & 1-\frac{u_{1n}-l_{1n}}{2m_{1n}}\\ 1-\frac{u_{21}-l_{21}}{2m_{21}} & 1 & \cdots & 1-\frac{u_{2n}-l_{2n}}{2m_{2n}}\\ \vdots & \vdots & \vdots & \vdots \\ 1-\frac{u_{n1}-l_{n1}}{2m_{n1}} & 1-\frac{u_{n2}-l_{n2}}{2m_{n2}} & \cdots & 1 \end{array}\right]$$

(7) $$Q=M\times E=\left[\begin{array}{cccc} m_{11} & m_{12} & \cdots & m_{1n}\\ m_{21} & m_{22} & \cdots & m_{2n}\\ \vdots & \vdots & \vdots & \vdots \\ m_{n1} & m_{n2} & \cdots & m_{nn} \end{array}\right]\left[\begin{array}{cccc} 1 & 1-\frac{u_{12}-l_{12}}{2m_{12}} & \cdots & 1-\frac{u_{1n}-l_{1n}}{2m_{1n}}\\ 1-\frac{u_{21}-l_{21}}{2m_{21}} & 1 & \cdots & 1-\frac{u_{2n}-l_{2n}}{2m_{2n}}\\ \vdots & \vdots & \vdots & \vdots \\ 1-\frac{u_{n1}-l_{n1}}{2m_{n1}} & 1-\frac{u_{n2}-l_{n2}}{2m_{n2}} & \cdots & 1 \end{array}\right]$$

$e_{ij}=\frac{u_{ij}-l_{ij}}{2m_{ij}}$ is the standard deviation rate, representing the degree of fuzziness of the expert evaluations. A larger value of $e_{ij}$ represents a greater degree of fuzziness and a greater degree of trustworthiness. Conversely, a lower value of $e_{ij}$ reflects less fuzziness and smaller reliability.

Step four involved solving the weights for each criterion. The final generated *W* = [*W*_1_, *W*_2_, …, *W_n_*]^T^ is the standard weight value obtained by calculating the *n*th root of all common factors according to Equations (8) and (9) and normalizing $\omega_{i}$ [111].

(8) $$\varpi_{i}={\left(\prod_{j=1}^{n}a_{ij}\right)}^{\frac{1}{n}},i=1,2,\cdots ,n$$

(9) $$\omega_{i}=\frac{\varpi_{i}}{\sum_{i=1}^{n}\varpi_{i}},i=1,2,\cdots ,n\;$$

### 2.5. Sensitivity Analysis

Although the triangular fuzzy hierarchical analysis method is more objective in determining the criteria weights of the indicators than the deterministic method, it is still influenced, to a lesser extent, by the decision-making experts. The OAT (once-at-a-time) sensitivity analysis was utilized, varying the weight value of one element at a time and examining the degree and regularity of the influence of single-factor weight changes on the change in findings while keeping the other factors as constant as possible [118]. 

The sensitivity analysis of the urban park siting criteria system requires a finite set of RPCs of discrete percentage change finite set RPCs with original base data. According to Ustaoglu and Aydinoglu (2020) [48], the RPC value for this study was defined as ±75%, representing a fluctuation in the standard original weights of −75% to +75%. The results of the fluctuations must refer to Equation (1) to ensure that the linear sum of all criteria weights is 1. Following Saatsaz et al. (2018) [119], the highest and lowest standard weight sensitivities were selected for sensitivity testing. Therefore, in this study, the sensitivity test was conducted separately for carbon storage (highest weight) and temperature (lowest weight). Similar to the procedure applied by Chen et al. (2010) [120], Selcuk (2013) [114], and Nyimbili and Erden (2020) [121], a 25% percentage change in IPC of the standard weights (highest and lowest) was consistently noted, indicating that the original weights of the highest and lowest standards fluctuated by 25% every time. Each weight fluctuated ±25%, ±50%, and ±75% for a total of six times, and the other weights needed to be adjusted following the corresponding standard weight ratios each time the weights changed, as follows [118,122]:

(10) $$W\left(C_{n},PC\right)=W\left(C_{n},0\right)+W\left(C_{n},0\right)\times PC\;1\leq n\leq 8\;$$

The initial weight value of the main criterion *C_n_* is denoted by *W*(*C_n_*, 0), and to ensure that the sum of the weights of all of the criteria is 1, the weights of the other criteria, *W*(*C_i_*, *PC*), had to be adjusted according to *W*(*C_n_*, *PC*), which was computed as follows:

(11) $$\;W\;\left(C_{i},\mathrm{PC}\right)=\left(1-W\left(C_{n},\mathrm{PC}\right)\right)\times W\left(C_{i},0\right)/\left(1-W\left(C_{n},0\right)\right)\;\left(i\neq n,1\leq n\leq 8\right)$$

where *W*(*C_i_*, 0) denotes the *i*th criterion’s initial weight value. Each time the weights of the primary criteria were altered according to the conditions in Equations (10) and (11), a new urban siting suitability map was generated, resulting in 12 operations and the creation of 12 urban park siting maps (available from the authors upon request). Tables in Section 3.3 show the changes in the weights and the final results of the sensitivity analysis.

## 3. Results

### 3.1. Weights Obtained by F-AHP Method

The F-AHP method was used to establish five criteria categories, physical factors, natural factors, environmental factors, accessibility factors, and human activity factors, and 13 sub-criteria for urban park site selection, including the following: elevation, slope, slope direction, precipitation, temperature, NDVI, heat-island effect, air pollution, carbon storage, distance to metro stations, distance to bus stops, distance to major roads, and distance to densely populated areas. Industry experts were invited to score the triangular fuzzy judgment matrix, and the average value was used to establish the weight of each factor. The median matrix was used for the triangular fuzzy judgment matrix consistency test. The factor weights were determined using the modified calculation of the triangular fuzzy hierarchy method [112]. Because of the large number of operations and steps, the calculations were written using MATLAB 2020 (for which the author can provide the specific code). The consistency test results and factor weights are illustrated in Table 3. The results showed that the carbon storage had the highest weight, and the temperature had the lowest weight. The environmental criteria of the carbon storage capacity, the NDVI, the heat-island effect, and the air pollution all had high weights, demonstrating that the quality of the ecological environment has a more significant effect on the location of urban parks. The population distribution in the built-up area criteria was second only to the environmental factors, and the denser the population distribution was, the more unfavorable the ecological environment of the park. For the park accessibility, the distances to metro stations/bus stops/main roads had similar weights. The elevation, the slope, and the aspect among the physical factors and the temperature and precipitation among the natural factors had lower weights compared to the other factors influencing the location of urban parks.

### 3.2. Nanjing City Park Site Selection Area

**Figure 5:**
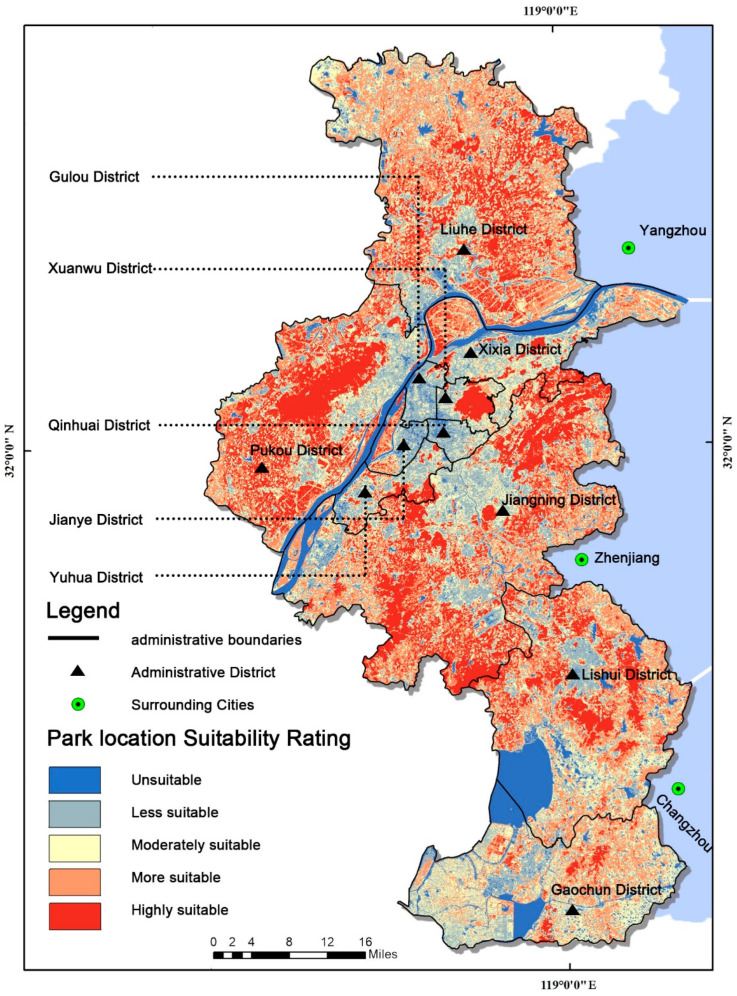
The final map of suitability site selection for urban parks in Nanjing, China.

In Nanjing, China a land suitability map for urban parks was created using the method that has been outlined above (Figure 5). The graph shows that the most suitable urban park site had the highest value, which is represented by dark blue, while the least suitable site had the lowest value, which is represented by orange. 

According to the spatial overlay analysis of the 13 criteria, the site selection area was classified as highly suitable, more suitable, moderately suitable, less suitable, and unsuitable. Overall, 21% (1401 km^2^) were highly suitable for urban park site selection, 28% (1844 km^2^) were more suitable, 24% (1578 km^2^) were moderately acceptable, 19% (1223 km^2^) were less suitable, and 8% (531 km^2^) were unsuitable for urban park site selection. The most suitable areas for urban park sites were concentrated in the west and east of Nanjing, where the ecological quality and accessibility were high and there were potential areas for urban park sites. The central core area contains most of the locations that are least suited for urban parks.

The final suitability map of Nanjing City from the different administrative districts showed that the Jiangning District (451 km^2^), Pukou District (319 km^2^), and Liuhe District (299 km^2^) were the top three political districts in terms of the highly suitable park area, and the Lishui District had the highest percentage of unsuitable park area of 135 km^2^. Approximately 21% of the area that was located mainly around ecological reserves, which are buffers of reservoirs and rivers, and near natural lands with high environmental value, including forests, agricultural lands, and natural vegetation, was classified as highly suitable.

By comparing the distribution of the existing urban parks and the highly suitable areas in Nanjing, it was found that 90% of the existing urban parks were located within qualified regions, indicating the high scientific validity of this study’s results. In addition, according to the data that is shown in Table 4, the existing urban park area in Nanjing was 183 km^2^, and the area that was highly suitable for urban parks in the study area covered 1401 km^2^, with nearly 1200 km^2^ being suitable for future urban park sites in Nanjing. Within the different administrative districts, the Jiangning District (431 km^2^), Pukou District (270 km^2^), and Liuhe District (296 km^2^) were the top three in terms of the gaps in urban park construction. Figure 5 shows that the northeast and southwest corners of the Jiangning District, the southwest of the Pukou District, and the south of the Liuhe District would be potential areas of future urban park sites in Nanjing. At the same time, the Qinhuai District, Xuanwu District, and Gulou District showed a negative urban park area gap, meaning they are not priority sites for future urban parks.

### 3.3. Results of Sensitivity Analysis

**Figure 6:**
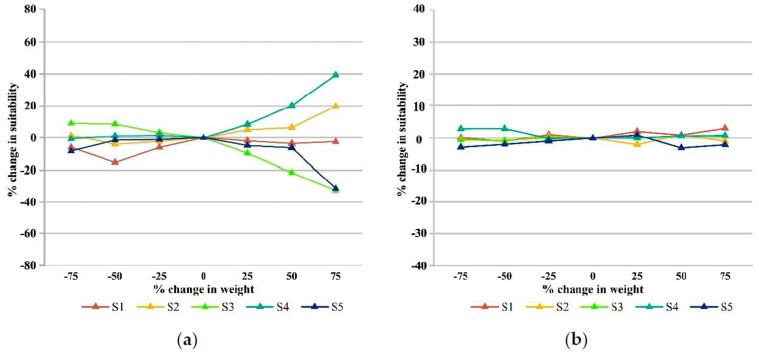
Results of sensitivity analysis of F-AHP applied to urban park site selection. (**a**) Sensitivity of the criterion ‘carbon storage’; (**b**) Sensitivity of the criterion ‘temperature’.

A sensitivity analysis of the urban park siting model was conducted using the OAT method. Table 5 shows the changes in the weights of the other criteria due to a ±75% change in the highest weight (carbon storage) and the lowest weight (temperature). In addition, the sensitivity results for the criteria with the highest weight (carbon storage) and the lowest weight (temperature) are reported in Table 6. Figure 6 summarizes the change in suitability (the percentage change from the initial value) that was obtained in the model. The study results showed that the criterion with the highest weight (carbon storage capacity) was more sensitive than the lower weight (temperature) criterion.

**Figure 7:**
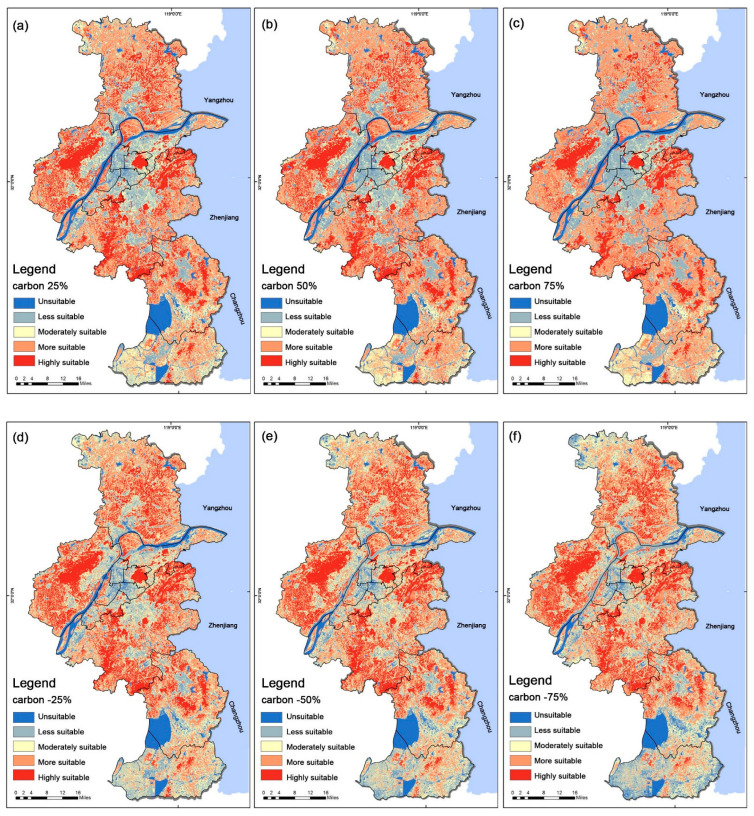
Results from sensitivity analysis from GIS for the criterion ‘carbon storage’ regarding About City Park Site Selection analysis: (**a**) Carbon 25%, (**b**) Carbon 50%, (**c**) Carbon 75%, (**d**) Carbon −25%, (**e**) Carbon −50%, (**f**) Carbon −75%.

In the model, ±75% change in the standard weight of carbon storage capacity caused −13% change in the S1 unsuitable class, 21% change in the S2 less suitable class, 41% change in the S3 moderately suitable class, 41% change in the S4 more suitable class and 23% change in the S5 highly suitable class. The changes in the standard weights of the carbon storage capacity significantly affected the moderately suitable S3 and the more suitable S4. The area of the S3 and the S5 reached a maximum reduction of 32.66% and 31.41%, respectively, when the weight was changed by +75%. Meanwhile, the S2 and the S4 reached a maximum increase of 19.74% and 39.44%, respectively. This shows that as the weight value increases, the area of highest suitability and medium suitability decreases significantly—most flow to the higher suitability areas and a small portion flow to the less suitable areas. Table 6 shows that when the weights decrease, the areas of S1, S4, and S5 decrease by 75%, with the S5 showing the most significant reduction of 8.28%. The carbon storage has the highest weight, so as its weight decreases, it will increase the weight values of the other factors, resulting in more pronounced fluctuations in the highly suitable zone (Figure 7).

**Figure 8:**
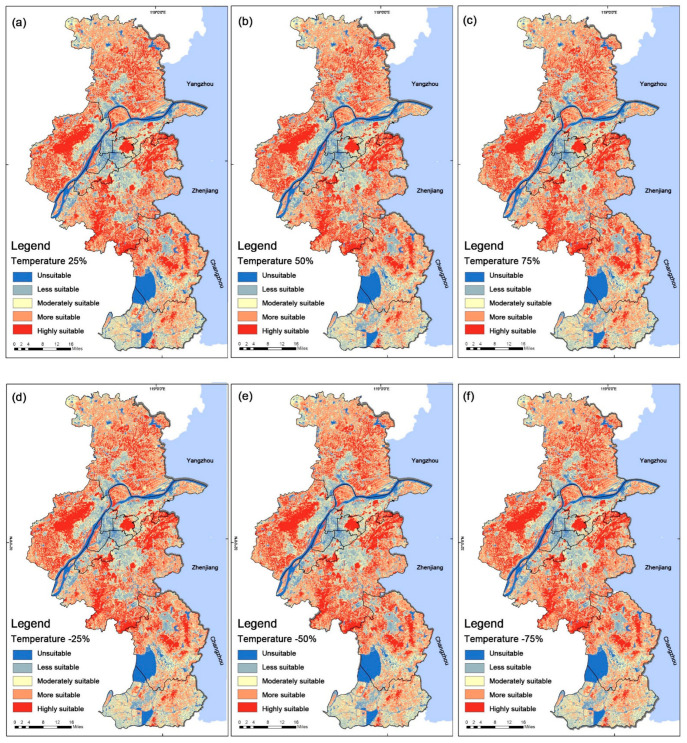
Results from sensitivity analysis from GIS for the criterion ‘temperature’ regarding About City Park Site Selection analysis: (**a**) Temperature 25%, (**b**) Carbon 50%, (**c**) Carbon 75%, (**d**) Temperature −25%, (**e**) Temperature −50%, (**f**) Temperature −75%.

The variation in suitability due to temperature criteria compared to carbon sequestration capacity ranged from 1% to 4%, with a slightly more significant effect of 4% minimum for moderate suitability S3 and a maximum impact of about 4.1% for the S1. The criterion for the minimum weight did not significantly affect the suitability distribution, so the changes that were brought about by the minimum weight relative to the maximum weight can be ignored (Figure 8).

## 4. Discussion

### 4.1. Discussion of Research Methods

An assessment system was developed in order to evaluate the land suitability of urban park sites within the study area. Four key categories and thirteen factor criteria were constructed scientifically by summarizing the previous finding. Fuzzy triangular numbers were used to calculate the factor weights while guaranteeing the scientific accuracy of the standardized factors’ spatial data sources. Sensitivity analysis was used in order to confirm the stability of the study results.

Unlike previous park site selection studies, we prospectively introduced fuzzy triangular numbers into GIS spatial overlay analysis and integrated fuzzy techniques into weight determination and GIS fuzzy function classification. This will provide new perspectives on incorporating the fuzzy methods into the evaluation of park site selection.

The research on land siting [123,124,125] commonly uses AHP hierarchical analysis in order to determine the weights of criteria as it is easier, faster, and more efficient. In the classic AHP approach, it is generally necessary to identify the relative significance of two factors through surveys and subjective evaluations. Consequently, it is easy to overlook the interactions between the diverse factors. Furthermore, the limitations of the AHP approach include insufficient knowledge of the area of concern, the reproducibility of results, and the subjectivity of the relative weights that are applied to the variables [48]. Several studies have acknowledged the AHP method’s drawbacks, including its inability to handle ambiguity and uncertainty in the decision-making process [40,126,127,128]. The fuzzy methods compensate for the drawbacks of the AHP approaches. As has been highlighted in the literature, fuzzy methods are more tractable and better equipped to deal with uncertainty in human decision making than the traditional AHP methods [129,130,131,132,133]. The triangular fuzzy AHP method uses fuzzy triangular numbers instead of the conventional AHP method of the 1~9 scale of importance values in order to establish a fuzzy judgment matrix. The range of values is called the confidence interval, which represents the decision maker’s confidence in the uncertainty of the problem being judged. The larger the range, the lower the confidence level of the large decision maker; the smaller the range, the higher the confidence level of the decision maker. Fuzzy AHP is a more advanced way to address the issue of uncertainty bias in expert judgments than traditional AHP. Specifically, the urban park site suitability map that was generated by the fuzzy technique may better explain the ideal site selection for urban parks. Among the weight values that were derived using the deterministic approach, criteria 1 and 2, criteria 7 and 8, and criteria 10, 11, and 12 have identical weight values (contact the author for the calculation results), making it impossible to distinguish the subtle importance and expert confidence in the different criteria. However, the criteria weight values that were determined by fuzzy triangulation in this research differed slightly (Table 3), and it is evident that the fuzzy technique yielded more accurate findings. Consequently, the map of the park site selection that was generated by the fuzzy technique can reflect the suitability characteristics of the park site selection area more accurately. The traditional questionnaire method that was used in this study is prone to inconsistency in the judgment matrix because of the large number of judgment matrix factors and the significant time cost of each expert’s survey during of the questionnaire [134,135]. According to Lyu et al. (2020) [136], the new questionnaire can be used in future studies in order to reduce the expert’s working time and to attenuate the effect of the matrix judgment inconsistencies on the results.

### 4.2. Limitations and Future Research Directions

Although the evaluation method combining GIS technology and the AHP technique is widely used to solve the problem of multi-objective land siting, the accuracy of the raster layers method for weighting and linear overlay is entirely dependent on the accuracy of the existing standard spatial data sources. Additionally, the criteria selected for the AHP index factor system are correlated, and the simple use of spatial linear weighting can cause certain factors with obvious limitations to be compensated for by the other criteria. The compromised factors can affect the limitations that can be expressed by the evaluator. Future studies should fully consider the variance in the criteria weights that diverse decision-makers’ preferences cause in this circumstance. In order to determine whether the evaluation outcomes of different study methods are consistent, we advise adopting other evaluation approaches, such as OWA techniques, artificial intelligence, and genetic algorithms, in future urban park site selection studies. 

The OAT is regarded as an appropriate sensitivity analysis technique for examining the effect of changes in a single factor on the evaluation outcomes. This study has examined the effects of the evaluation outcomes on the two criteria with the highest and lowest weights. Although abnormal criteria were not discovered, the sensitivity monitoring scope should be as expansive as feasible in future urban site selection studies in order to rule out the effect of particular standards on the evaluation outcomes. According to Talukdar et al. (2022) [137], various sensitivity testing methods, such as global sensitivity analysis using the Morris model, random-forest-dependent sensitivity analysis, and Pearson’s correlation coefficient, can be applied simultaneously in the fuzzy reliability test in order to compare the effects of independent factors of the target variables. In this study, we generated a site map of Nanjing’s urban parks, which categorizes the land as highly suitable, more suitable, moderately suitable, less suitable, and unsuitable areas. Overall, the western and eastern parts of Nanjing have large amounts of land that are suitable for park construction. The Xuanwu District features the highest percentage of land that is suitable for park construction, which is closely linked to its unique geomorphology. The largest royal garden lake in China, Xuanwu Lake, and the popular national 5A scenic area, Zhongshan Mountain, are both located within the Xuanwu District, which boasts unique natural ecological environments. 

The study results in Table 4 show that the percentage of land that is suitable for park sites in the Gulou District is about 4%, but the area of land that is occupied by competing parks is 11%, which means that some of the park sites are not suitable locations. Unsurprisingly, the area is adjacent to Xuanwu Lake and hosts some of the oldest and most-central parts of Nanjing’s old town. Much of the park’s planning was already completed decades ago and did not rely entirely on scientific techniques for processing geographic information [138]. Hence, subjective factors of planning decision-makers and socio-economic criteria had more influence [36]. Table 4 shows that the area of suitable park construction in three administrative districts, the Qinhuai District, Xuanwu District, Gulou District, and Jianye District, is close to saturation. Therefore, park construction sites in the Nanjing City Park Development Plan (2017–2035) must avoid these administrative districts. The Jiangning District, Pukou District, and Liuhe District, as city suburbs, currently have a gap of more than 270 km^2^ of suitable area that is for park construction (Table 4). The region boasts a large amount of natural land of high ecological value and developed road access, making it a priority site for the future construction of Nanjing Park. Urban park types come in a wide variety, with clear distinctions regarding their objectives, their physical attributes, and their geographic locations. The criteria system that has been created in this study was based on the similarities that can be found in urban parks, but it did not consider the unique characteristics of certain types of urban parks. The future in-depth study of urban parks should address these limitations, focusing on the standard system for various urban parks. For instance, urban regions with better ecological conditions and more natural vegetation can be used as construction sites for environmental parks and areas with great accessibility and proximity to residential areas can be used as construction sites for community parks. It is beneficial for the decision-making authorities in this case study area to develop strategies that are suitable for urban park development by analyzing various forms of urban parks. The model that has been tested in this study can be applied to other national regions as well.

## 5. Conclusions

This paper has proposed the combination of fuzzy triangular sets, fuzzy AHP, and GIS for land-siting analysis of urban parks in the Nanjing area. The triangular fuzzy hierarchical analysis method F-AHP–GIS is more advanced than the traditional AHP–GIS method because it uses fuzzy set theory in order to better deal with unclear problems. We introduced fuzzy triangular numbers in order to construct a judgment matrix, which improves the error between the two-two factor comparisons, weakens the human subjective preferences, and enhances the performance of the multi-criteria decision model for park city site selection.

The final urban park site map was created using GIS spatial overlay technology and was classified into highly suitable, more suitable, moderately suitable, less suitable, and unsuitable areas. About 21.30% (1401 km^2^) of the area was highly suitable, about 28.04% (1844 km^2^) was more suitable, about 23.99% (1578 km^2^) was moderately suitable, about 18.60% (1223 km^2^) was less suitable, and about 8.07% (531 km^2^) was unsuitable.

The results of the OAT sensitivity analysis that was based on the highest and lowest weights showed that the high weighted carbon storage factor had a significant effect on the suitability map, with the fluctuating area of the S3 and the S4 regions reaching 41%. From the above data, we have concluded that the highest weighting factor that is used for urban park suitability site selection significantly influences the assessment results. Therefore, the carbon stock data sources in the article were selected from the 2018 30 m resolution Chinese land use remote sensing data and the Stanford University InVEST model for analysis, ensuring the reliability of the information sources and improving the stability of the research results.

In general, our study of the siting of established urban parks in Nanjing, China, found that it appropriately considered the physical, natural, environmental, accessibility, and human activity factors in the siting of parks. Nevertheless, the study has revealed that some established urban park sites are incompatible with the urban park’s suitability map. Consequently, the urban planning department of the Nanjing area and government policymakers should re-evaluate these locations.

## Figures and Tables

**Table 1:** The evaluation criteria for city park site selection with fuzzy membership function.

Main Criteria	Sub-Criteria	Upper-Limit	Lower-Limit	Function Type	References
Physical	Elevation	448.0	−122	Linear (decreasing)	Ustaoglu and Aydinoglu (2020); Luan et al. (2021) [48,86]
	Slope	64.0	0	Linear (decreasing)	Ustaoglu and Aydinoglu (2020); Luan et al. (2021) [48,86]
	Aspect	225	10	Gaussian	Rahimi et al. (2020) [103]
Natural	Precipitation	1570.1	1271.1	Linear (decreasing)	Pussel and Li (2019); Ronizi et al. (2020); Zabihi et al. (2020) [40,104,105]
	Temperature	17.30	15.24	Linear (increasing)	Pussel and Li (2019); Mokarram et al. (2020); Zabihi et al. (2020) [40,104,105]
Environmental	NDVI	1	0	Linear (increasing)	Pussel and Li (2019); Pakfetrat et al. (2020); Smith et al. (2021) [30,105,106]
	Heat-island effect	49.9	14.2	Linear (decreasing)	Zhang et al. (2019); Smith et al. (2021) [35,106]
	Air pollution	36.2	24.4	Linear (decreasing)	Neema and Ohgai (2010); Pussel and Li (2019); Zhang et al. (2019) [31,35,105]
	Carbon storage	25.9	0.1	Linear (increasing)	Gratani et al. (2016); Shadman et al. (2022) [107,108]
Accessible	Distance to metro stations	36,430	0	Linear (decreasing)	Zhang et al. (2013); Pussel and Li (2019) [85,105]
	Distance to bus stops	13,810.9	0	Linear (decreasing)	Pussel and Li (2019) [105]
	Distance to major roads	6340.2	0	Linear (decreasing)	Zhang et al. (2013); Wang and Zhang (2014) [85,109]
Human activities	Distance to densely populated areas	240.1	0	Linear (decreasing)	Neema and Ohgai (2010); Zhang et al. (2013); Zhang et al. (2019); Rahimi et al. (2020); Smith et al. (2021) [31,35,85,103,106]

**Table 2:** Fuzzy comparison measures [115,116].

Linguistic Scale of Importance	Fuzzy Number	Triangular Fuzzy Scale	Reciprocal Fuzzy Scale
Equally important	1	(1, 1, 1)	(1, 1, 1)
Moderately important	3	(2, 3, 4)	(1/4, 1/3, 1/2)
Strongly important	5	(4, 5, 6)	(1/6, 1/5, 1/4)
Very strongly important	7	(6, 7, 8)	(1/8, 1/7, 1/6)
Absolutely more important	9	(9, 9, 9)	(1/9, 1/9, 1/9)
Intermediate value	2	(1, 2, 3)	(1/3, 1/2, 1)
	4	(3, 4, 5)	(1/5, 1/4, 1/3)
	6	(5, 6, 7)	(1/7, 1/6, 1/5)
	8	(7, 8, 9)	(1/9, 1/8, 1/7)

**Table 3:** Calculated fuzzy aggregated decision matrix of criteria and the normalized priority weight.

Layers	Elevation	Slope	Aspect	Precipitation	Temperature	NDVI	Heat-Island Effect	Pollution	Carbon	Metro Stations	Bus Station	Road	Population
Elevation	(1,1,1)	(1,1,2)	(1,2,2)	(1,3,4)	(1,3,4)	(0.14,0.17,0.2)	(0.17,0.2,0.25)	(0.17,0.2,0.25)	(0.13,0.14,0.14)	(0.25,0.33,0.5)	(0.25,0.33,0.5)	(0.2,0.33,0.33)	(0.2,0.25,0.33)
Slope	(0.5,1,1)	(1,1,1)	(1,2,2)	(2,3,3)	(2,3,3)	(0.14,0.17,0.2)	(0.17,0.2,0.25)	(0.17,0.2,0.25)	(0.13,0.14,0.17)	(0.25,0.33,0.5)	(0.25,0.33,0.5)	(0.25,0.33,0.33)	(0.2,0.25,0.33)
Aspect	(0.5,0.5,1)	(0.5,0.5,1)	(1,1,1)	(1,2,2)	(1,2,2)	(0.13,0.14,0.17)	(0.14,0.17,0.2)	(0.14,0.17,0.2)	(0.11,0.13,0.14)	(0.2,0.25,0.33)	(0.2,0.25,0.33)	(0.17,0.25,0.33)	(0.17,0.2,0.25)
Precipitation	(0.25,0.33,1)	(0.33,0.33,0.5)	(0.5,0.5,1)	(1,1,1)	(0.5,1,1)	(0.11,0.13,0.14)	(0.13,0.14,0.17)	(0.13,0.14,0.17)	(0.11,0.11,0.13)	(0.17,0.2,0.25)	(0.17,0.2,0.25)	(0.17,0.2,0.2)	(0.14,0.17,0.2)
Temperature	(0.25,0.33,1)	(0.33,0.33,0.5)	(0.5,0.5,1)	(1,1,2)	(1,1,1)	(0.11,0.13,0.14)	(0.13,0.14,0.17)	(0.13,0.14,0.17)	(0.11,0.11,0.13)	(0.17,0.2,0.25)	(0.17,0.2,0.25)	(0.17,0.2,0.2)	(0.14,0.17,0.2)
NDVI	(5,6,7)	(5,6,7)	(6,7,8)	(7,8,9)	(7,8,9)	(1,1,1)	(1,2,3)	(1,2,4)	(0.33,0.5,1)	(3,4,5)	(3,4,5)	(3,4,4)	(1,3,4)
Heat-Island Effect	(4,5,6)	(4,5,6)	(5,6,7)	(6,7,8)	(6,7,8)	(0.33,0.5,1)	(1,1,1)	(1,1,2)	(0.25,0.33,0.5)	(1,3,4)	(1,3,4)	(2,3,3)	(1,2,2)
Pollution	(4,5,6)	(4,5,6)	(5,6,7)	(6,7,8)	(6,7,8)	(0.25,0.5,1)	(0.5,1,1)	(1,1,1)	(0.33,0.33,0.5)	(1,3,3)	(1,3,3)	(2,3,3)	(1,2,3)
Carbon	(7,7,8)	(6,7,8)	(7,8,9)	(8,9,9)	(8,9,9)	(1,2,3)	(2,3,4)	(2,3,3)	(1,1,1)	(4,5,6)	(4,5,6)	(4,5,5)	(3,4,5)
Metro Station	(2,3,4)	(2,3,4)	(3,4,5)	(4,5,6)	(4,5,6)	(0.2,0.25,0.33)	(0.25,0.33,1)	(0.33,0.33,1)	(0.17,0.2,0.25)	(1,1,1)	(1,1,2)	(0.5,1,1)	(0.33,0.5,1)
Bus Station	(2,3,4)	(2,3,4)	(3,4,5)	(4,5,6)	(4,5,6)	(0.2,0.25,0.33)	(0.25,0.33,1)	(0.33,0.33,1)	(0.17,0.2,0.25)	(0.5,1,1)	(1,1,1)	(0.5,1,1)	(0.33,0.5,1)
Road	(3,3,5)	(3,3,4)	(3,4,6)	(5,5,6)	(5,5,6)	(0.25,0.25,0.33)	(0.33,0.33,0.5)	(0.33,0.33,0.5)	(0.2,0.2,0.25)	(1,1,2)	(1,1,2)	(1,1,1)	(0.33,0.5,1)
Population	(3,4,5)	(3,4,5)	(4,5,6)	(5,6,7)	(5,6,7)	(0.25,0.33,1)	(0.5,0.5,1)	(0.33,0.5,1)	(0.2,0.25,0.33)	(1,2,3)	(1,2,3)	(1,2,3)	(1,1,1)
Weight	0.038825	0.036223	0.023650	0.014442	0.014364	0.148227	0.120379	0.120349	0.176840	0.071047	0.071124	0.070274	0.094254
Rank	9	10	11	12	13	2	3	4	1	7	6	8	5
Consistency Check	*λ_max_* = 13.544; *CI* = 0.045; *CR* = 0.029; Consistency check passed.												

**Table 4:** The ecological suitability of the site area for urban parks in Nanjing, China.

District	Classes of the Urban Parks					Existing Urban Parks	
	S1	S2	S3	S4	S5	Area	Difference
Jianye	20 km^2^	26 km^2^	12 km^2^	13 km^2^	7 km^2^	2 km^2^	6 km^2^
Lishui	135 km^2^	169 km^2^	238 km^2^	334 km^2^	182 km^2^	27 km^2^	155 km^2^
Qinhuai	6 km^2^	30 km^2^	11 km^2^	2 km^2^	0 km^2^	1 km^2^	0 km^2^
Xuanwu	1 km^2^	21 km^2^	18 km^2^	8 km^2^	28 km^2^	30 km^2^	−2 km^2^
Qixia	56 km^2^	97 km^2^	103 km^2^	80 km^2^	54 km^2^	33 km^2^	21 km^2^
Gulou	8 km^2^	34 km^2^	9 km^2^	1 km^2^	2 km^2^	6 km^2^	−4 km^2^
Gaochun	97 km^2^	180 km^2^	273 km^2^	209 km^2^	34 km^2^	3 km^2^	32 km^2^
Yuhua	11 km^2^	47 km^2^	33 km^2^	17 km^2^	24 km^2^	11 km^2^	13 km^2^
Jiangning	71 km^2^	261 km^2^	335 km^2^	444 km^2^	451 km^2^	20 km^2^	431 km^2^
Pukou	48 km^2^	135 km^2^	184 km^2^	222 km^2^	319 km^2^	49 km^2^	270 km^2^
Liuhe	77 km^2^	224 km^2^	361 km^2^	514 km^2^	299 km^2^	3 km^2^	296 km^2^
Total	531 km^2^ (8%)	1223 km^2^ (19%)	1578 km^2^ (24%)	1844 km^2^ (28%)	1401 km^2^ (21%)	183 km^2^	1217 km^2^

Note: Class S1—unsuitable. Class S2—less suitable. Class S3—moderately suitable. Class S4—more suitable. Class S5—highly suitable.

**Table 5:** Results on the variation of weight fluctuations of the ‘carbon storage’ and ‘temperature’ criteria for the siting analysis of urban parks.

Change (%)	Weight Value (Carbon Storage)												
	Elevation	Slope	Aspect	Precipitation	Temperature	NDVI	Heat-Island Effect	Pollution	Carbon	Metro Stations	Bus Station	Road	Population
75	0.0326	0.0304	0.0198	0.0121	0.0120	0.1243	0.1010	0.1010	0.3095	0.0596	0.0597	0.0590	0.0791
50	0.0347	0.0323	0.0211	0.0129	0.0128	0.1323	0.1074	0.1074	0.2653	0.0634	0.0635	0.0627	0.0841
25	0.0367	0.0343	0.0224	0.0137	0.0136	0.1403	0.1139	0.1139	0.2211	0.0672	0.0673	0.0665	0.0892
0	0.0388	0.0362	0.0237	0.0144	0.0144	0.1482	0.1204	0.1203	0.1768	0.0710	0.0711	0.0703	0.0943
−25	0.0409	0.0382	0.0249	0.0152	0.0151	0.1562	0.1268	0.1268	0.1326	0.0749	0.0749	0.0740	0.0993
−50	0.0430	0.0401	0.0262	0.0160	0.0159	0.1641	0.1333	0.1333	0.0884	0.0787	0.0788	0.0778	0.1044
−75	0.0451	0.0421	0.0275	0.0168	0.0167	0.1721	0.1398	0.1397	0.0442	0.0825	0.0826	0.0816	0.1094
**Change (%)**	**Weight Value (Temperature)**												
	**Elevation**	**Slope**	**Aspect**	**Precipitation**	**Temperature**	**NDVI**	**Heat-Island Effect**	**Pollution**	**Carbon**	**Metro Stations**	**Bus Station**	**Road**	**Population**
75	0.0384	0.0358	0.0234	0.0143	0.0251	0.1466	0.1191	0.1190	0.1749	0.0703	0.0703	0.0695	0.0932
50	0.0385	0.0360	0.0235	0.0143	0.0215	0.1471	0.1195	0.1195	0.1756	0.0705	0.0706	0.0698	0.0936
25	0.0387	0.0361	0.0236	0.0144	0.0180	0.1477	0.1199	0.1199	0.1762	0.0708	0.0709	0.0700	0.0939
0	0.0388	0.0362	0.0237	0.0144	0.0144	0.1482	0.1204	0.1203	0.1768	0.0710	0.0711	0.0703	0.0943
−25	0.0390	0.0364	0.0237	0.0145	0.0108	0.1488	0.1208	0.1208	0.1775	0.0713	0.0714	0.0705	0.0946
−50	0.0391	0.0365	0.0238	0.0145	0.0072	0.1493	0.1213	0.1212	0.1781	0.0716	0.0716	0.0708	0.0949
−75	0.0392	0.0366	0.0239	0.0146	0.0036	0.1498	0.1217	0.1217	0.1788	0.0718	0.0719	0.0710	0.0953

**Table 6:** Results from sensitivity analysis from fuzzy-AHP methods for the criterion ‘carbon storage’ and ‘temperature’ regarding About City Park Site Selection analysis.

Change (%)	Suitability (km^2^)					Change in Suitability(%)				
	S1	S2	S3	S4	S5	S1	S2	S3	S4	S5
	Carbon storage									
75	517.58	1464.48	1062.57	2570.81	960.67	−2.53%	19.74%	−32.66%	39.44%	−31.41%
50	511.41	1301.14	1238.25	2213.31	1312.00	−3.69%	6.38%	−21.52%	20.05%	−6.32%
25	520.00	1285.00	1426.00	2013.00	1331.00	−2.07%	5.06%	−9.62%	8.41%	−4.97%
0	531.02	1223.07	1577.81	1843.64	1400.58	0.00%	0.00%	0.00%	0.00%	0.00%
−25	498.44	1193.38	1628.87	1871.49	1383.93	−6.13%	−2.43%	3.24%	1.51%	−1.19%
−50	449.61	1172.32	1711.49	1864.83	1377.85	−15.33%	−4.15%	8.47%	1.15%	−1.62%
−75	499.34	1242.59	1720.72	1828.80	1284.66	−5.96%	1.60%	9.06%	−0.80%	−8.28%
	Temperature									
75	547.46	1210.51	1587.90	1858.94	1371.30	3.10%	−1.03%	0.64%	0.83%	−2.09%
50	536.13	1235.71	1589.14	1857.65	1357.49	0.96%	1.03%	0.72%	0.76%	−3.08%
25	542.36	1197.86	1576.75	1845.02	1414.13	2.14%	−2.06%	−0.07%	0.07%	0.97%
0	531.02	1223.07	1577.81	1843.64	1400.58	0.00%	0.00%	0.00%	0.00%	0.00%
−25	537.02	1231.01	1578.77	1842.15	1387.16	1.13%	0.65%	0.06%	−0.08%	−0.96%
−50	525.49	1210.94	1566.72	1899.15	1373.82	−1.04%	−0.99%	−0.70%	3.01%	−1.95%
−75	531.74	1218.28	1567.58	1897.93	1360.58	0.14%	−0.39%	−0.65%	2.94%	−2.86%

## Data Availability

Data are readily available at request.
